# Supplementary material for: Efficient Propagation of Circulating Tumor Cells: A First Step for Probing Tumor Metastasis
Source: Cancers (Basel). 2020 Sep 28;12(10):2784. doi: 10.3390/cancers12102784 (PMC7599955; doi:10.3390/cancers12102784)
Supplement: Supplementary file 1 [file cancers-12-02784-s001.pdf]

cancers

Supplementary Material: Efficient Propagation of Circulating Tumor Cells: a First Step for Probing Tumor Metastasis

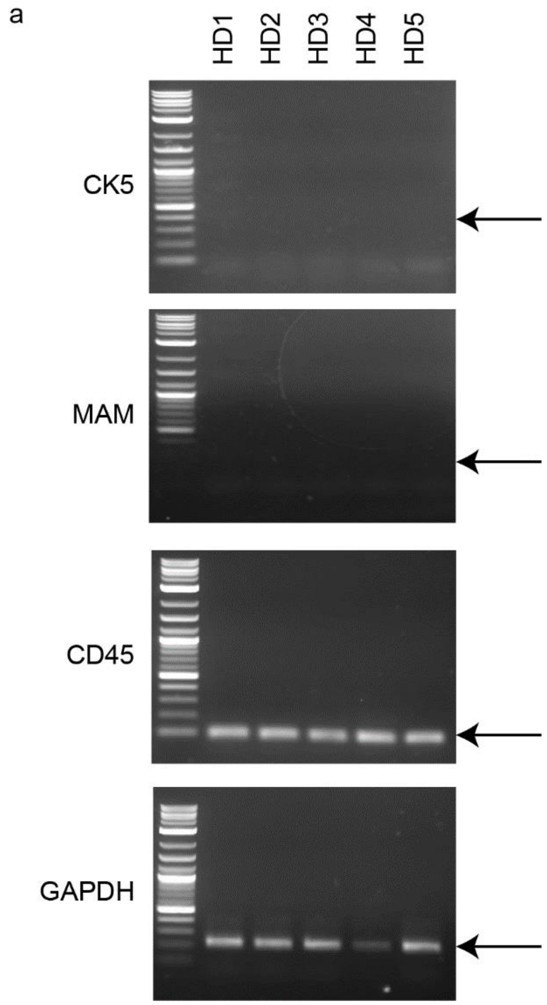

Figure S1. PCR of markers in healthy donors. a, Original gel images of PCR products for epithelial (cytokeratin 5, CK5), breast (mammaglobin, MAM), leukocytes (CD45), and housekeeping gene GAPDH amplified from RNA derived from healthy donors. Black arrows indicate expected PCR product band size.

a

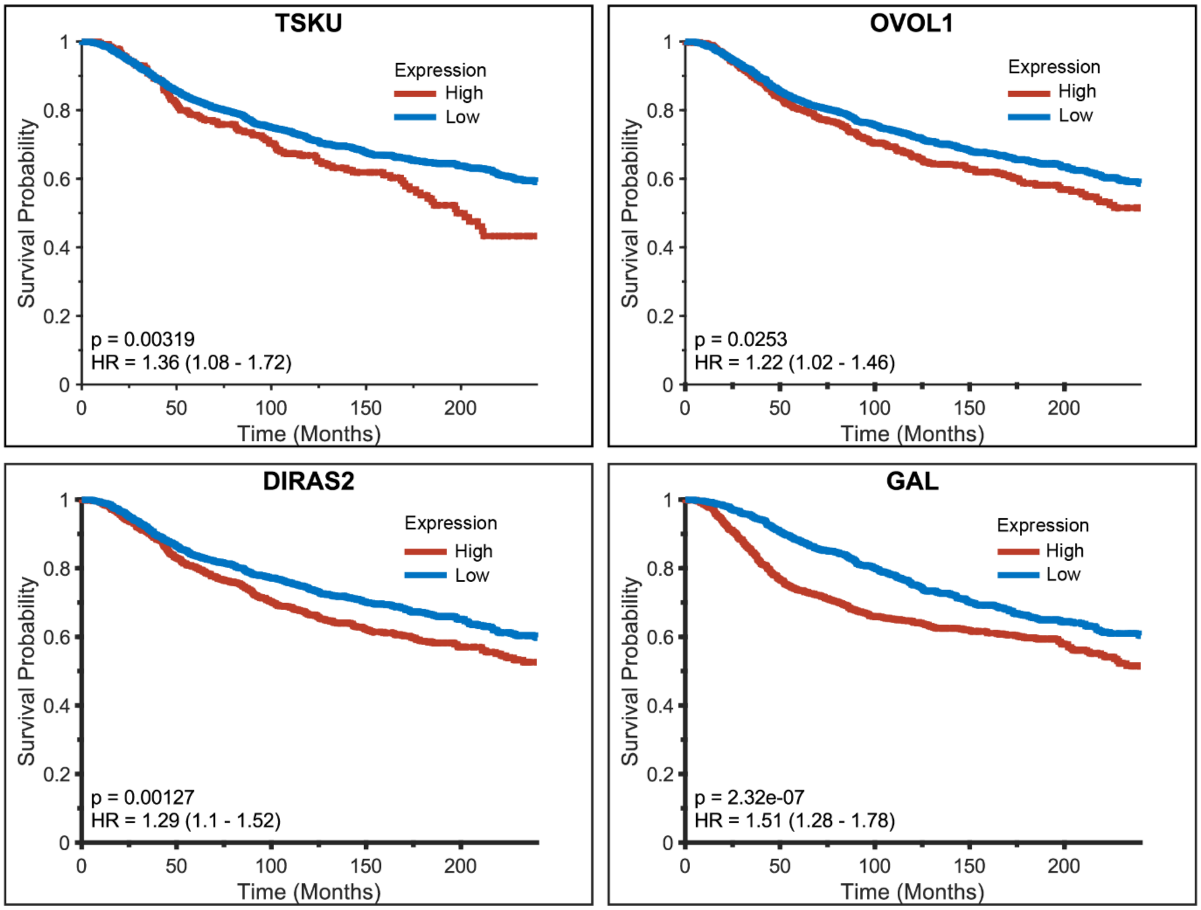

Figure S2. Kaplan-Meier analysis of representative upregulated genes from RNA-seq using METABRIC. METABRIC is a publicly accessible database of 1904 primary breast tumors with gene expression data. Shown here are examples of the top upregulated genes from our RNA-seq data that show high expression correlated with lower overall survival in the METABRIC study.

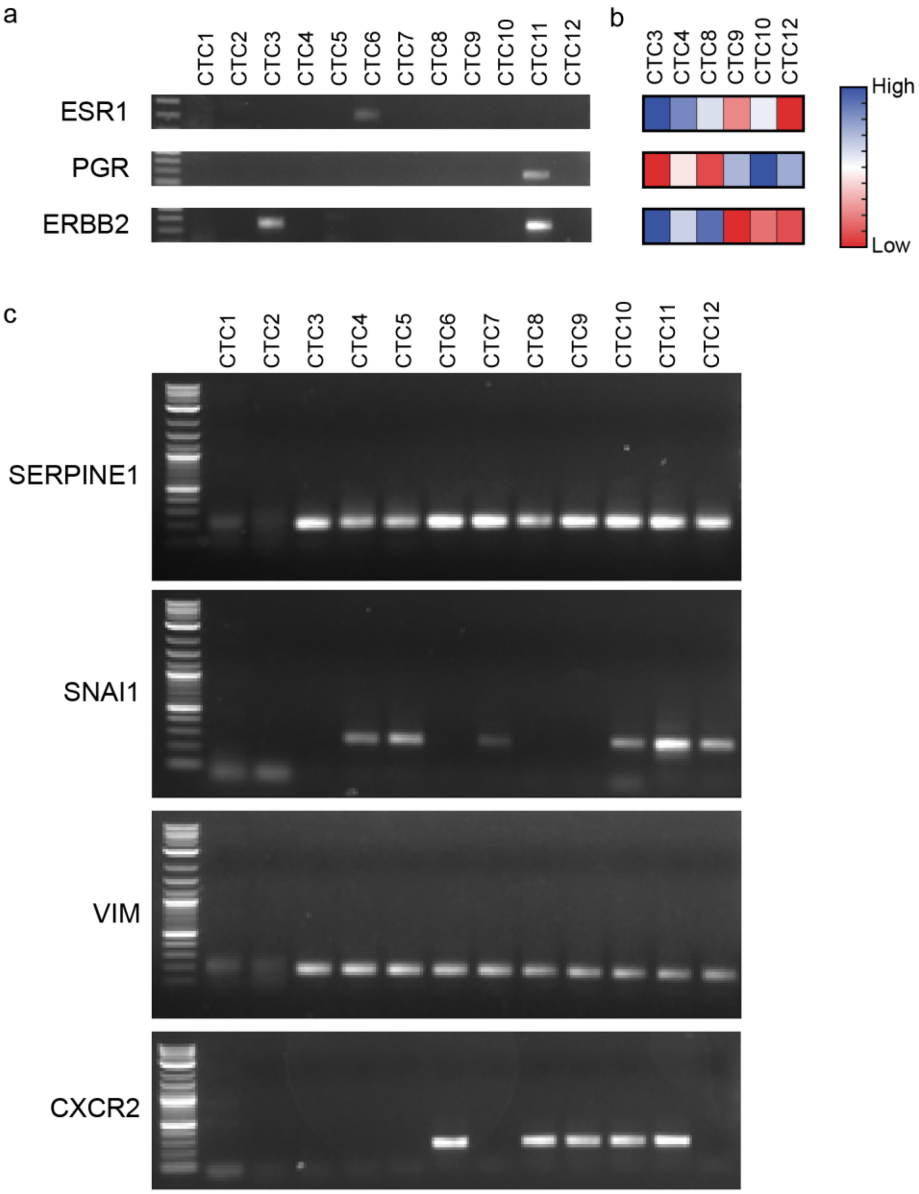

Figure S3. PCR of Estrogen, Progesterone, and HER2 receptors in CTCs. **a**, RT-PCR was performed on the twelve CTCs to determine receptor status. **b,** A corresponding heatmap depicting RNA-seq reads of 6 CTC samples. **c**, RT-PCR results of various genes using cDNA made from CTC samples. See **Table S4** for primers used.

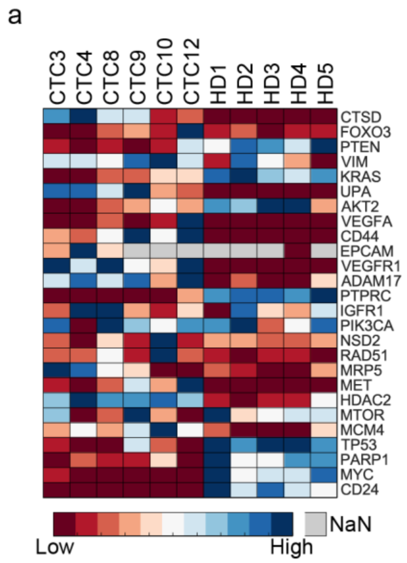

Figure S4. Similar "CTC" signature as compared to Hensler et al., 2016. a, RNA-seq results for selected markers as reported in Figure 1b from Hensler et al., 2016 [18]. This study used gene expression profiling to identify 27 mRNA transcripts that were enriched in CTCs compared to healthy controls, which was dubbed a "CTC" signature.

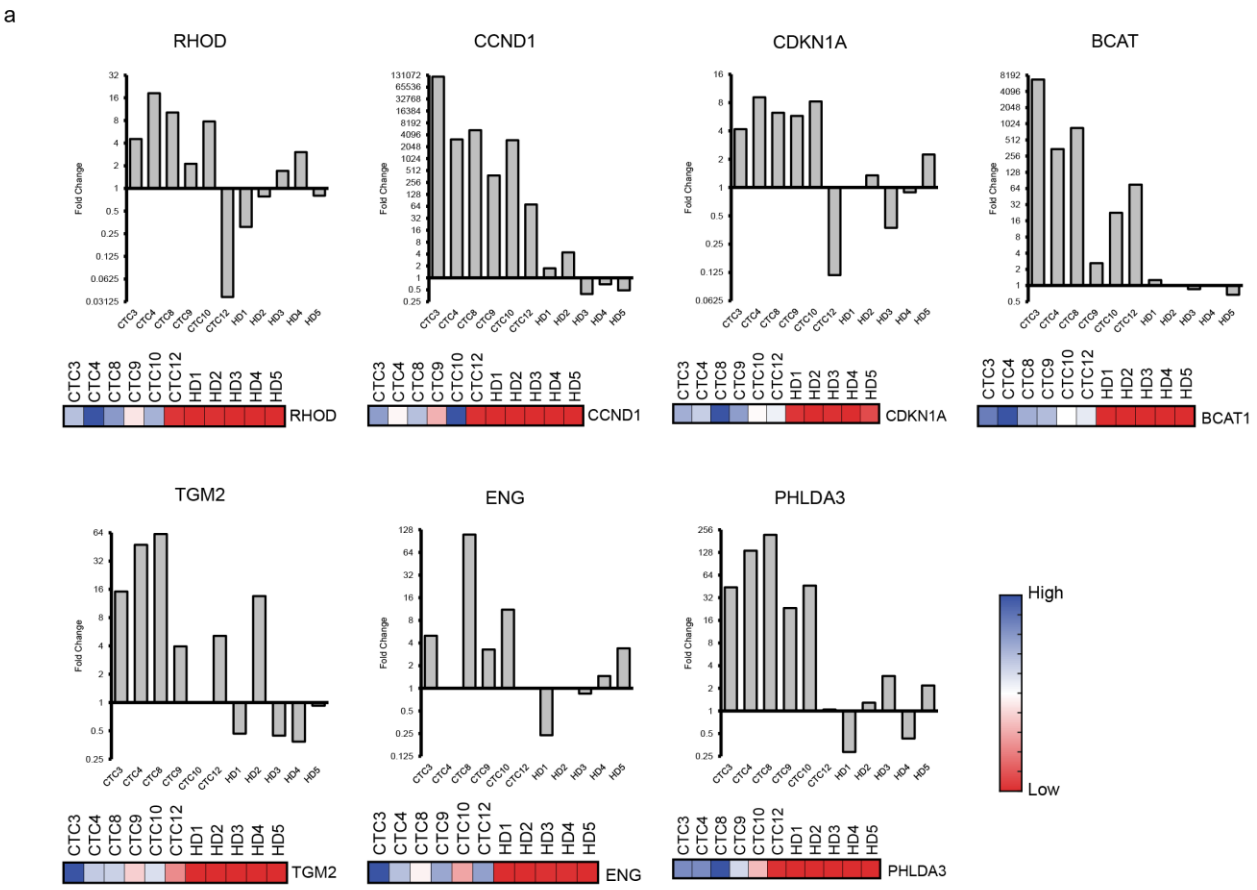

Figure S5. Quantitative RT-PCR validation of RNA samples using select genes derived from Fig. 3d-g. a, qRT-PCR was performed on seven genes involved in cancer-associated pathways to validate RNA-seq data. Heatmaps underneath each bar graph represent the correlating RNA-seq data.

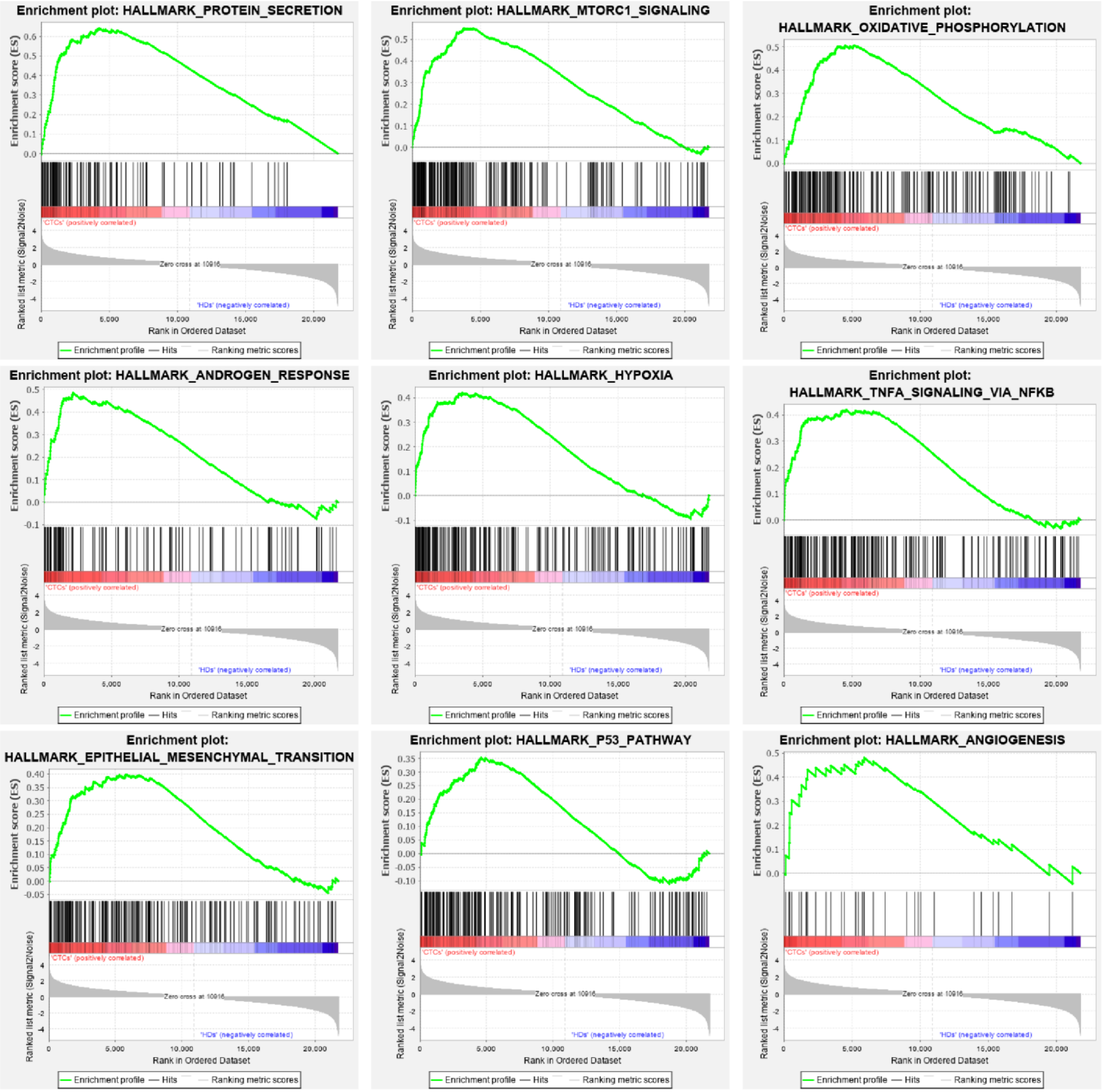

Figure S6. Enrichment plots from GSEA analysis on CTCs. Representative enrichment plots for the top 21 Hallmarks genesets that were enriched. Nominal p-value < 0.05 and FDR < 0.25 were used to determine statistical significance.

Table S1. Comprehensive list of all statistically significant KEGG pathways enriched in our RNA-sequencing data. A total of 52 KEGG pathways were enriched in CTCs compared to HDs.

	Pathway	N	DE	adj.p
path:hsa01100	Metabolic pathways	1487	553	4.35E-21
path:hsa04142	Lysosome	128	80	6.96E-16
path:hsa03010	Ribosome	153	85	1.49E-12
path:hsa04145	Phagosome	150	72	1.24E-06
path:hsa05200	Pathways in cancer	530	194	5.93E-06
path:hsa04216	Ferroptosis	40	27	1.20E-05
path:hsa05418	Fluid shear stress and atherosclerosis	138	65	1.71E-05
path:hsa05323	Rheumatoid arthritis	90	47	2.43E-05
path:hsa04640	Hematopoietic cell lineage	96	49	3.30E-05
path:hsa04621	NOD-like receptor signaling pathway	180	78	7.14E-05
path:hsa00480	Glutathione metabolism	55	32	0.000116
path:hsa01200	Carbon metabolism	117	55	0.000188
path:hsa05165	Human papillomavirus infection	330	125	0.000246
path:hsa05202	Transcriptional misregulation in cancer	186	78	0.000346
path:hsa05120	Epithelial cell signaling in Helicobacter pylori infection	70	37	0.000384
path:hsa05130	Pathogenic Escherichia coli infection	202	83	0.000431
path:hsa04068	FoxO signaling pathway	131	59	0.000437
path:hsa04066	HIF-1 signaling pathway	109	51	0.000556
path:hsa00520	Amino sugar and nucleotide sugar metabolism	48	28	0.000567
path:hsa00514	Other types of O-glycan biosynthesis	46	27	0.00073
path:hsa04115	p53 signaling pathway	72	37	0.000917
path:hsa05222	Small cell lung cancer	92	44	0.001341
path:hsa05146	Amoebiasis	102	47	0.0023
path:hsa00310	Lysine degradation	61	32	0.002387
path:hsa04510	Focal adhesion	198	79	0.002759
path:hsa05219	Bladder cancer	41	24	0.002787
path:hsa01230	Biosynthesis of amino acids	75	37	0.003069
path:hsa05134	Legionellosis	57	30	0.004138
path:hsa03320	PPAR signaling pathway	76	37	0.004474
path:hsa04512	ECM-receptor interaction	88	41	0.006059
path:hsa04978	Mineral absorption	58	30	0.006403
path:hsa05230	Central carbon metabolism in cancer	69	34	0.007052
path:hsa01212	Fatty acid metabolism	56	29	0.008446
path:hsa04061	Viral protein interaction with cytokine and cytokine receptor	98	44	0.009508
path:hsa05163	Human cytomegalovirus infection	223	85	0.009549
path:hsa04151	PI3K-Akt signaling pathway	354	125	0.011885
path:hsa00100	Steroid biosynthesis	20	14	0.012708
path:hsa00010	Glycolysis / Gluconeogenesis	68	33	0.013444
path:hsa04926	Relaxin signaling pathway	129	54	0.014308
path:hsa04540	Gap junction	88	40	0.014878
path:hsa00970	Aminoacyl-tRNA biosynthesis	66	32	0.017667
path:hsa04979	Cholesterol metabolism	50	26	0.019233
path:hsa05144	Malaria	50	26	0.019233

path:hsa04015	Rap1 signaling pathway	210	79	0.028766
path:hsa05131	Shigellosis	236	87	0.030389
path:hsa00531	Glycosaminoglycan degradation	19	13	0.033892
path:hsa05167	Kaposi sarcoma-associated herpesvirus infection	186	71	0.03718
path:hsa04933	AGE-RAGE signaling pathway in diabetic complications	100	43	0.037896
path:hsa04064	NF-kappa B signaling pathway	103	44	0.038121
path:hsa00270	Cysteine and methionine metabolism	49	25	0.038433
path:hsa04218	Cellular senescence	158	62	0.038751
path:hsa04612	Antigen processing and presentation	69	32	0.049272

Table S2. Comprehensive list of all statistically significant GO-processes enriched in RNA-sequencing data. A list of statistically significant (adjusted p-value < 0.05) GO-terms were generated.

	Term	N	DE	adj.p
GO:0008150	biological_process	18367	5578	8.58E-123
GO:0050896	response to stimulus	9207	3061	7.96E-79
GO:0051179	localization	6696	2350	6.67E-78
GO:0009987	cellular process	16698	5011	1.60E-77
GO:0002376	immune system process	3152	1277	1.68E-76
GO:0006810	transport	5160	1843	8.22E-63
GO:0051234	establishment of localization	5285	1876	7.29E-62
GO:0006955	immune response	2240	922	3.51E-56
GO:0071840	cellular component organization or biogenesis	6656	2240	1.70E-54
GO:0016043	cellular component organization	6472	2181	4.80E-53
GO:0065007	biological regulation	12632	3848	5.02E-51
GO:0051716	cellular response to stimulus	7518	2418	1.33E-41
GO:0001775	cell activation	1423	608	5.33E-41
GO:1901564	organonitrogen compound metabolic process	6986	2261	5.74E-40
GO:0050794	regulation of cellular process	10896	3326	8.01E-40
GO:0050789	regulation of biological process	11951	3602	3.20E-39
GO:0048518	positive regulation of biological process	6218	2038	1.34E-38
GO:0065008	regulation of biological quality	4048	1404	1.18E-36
GO:0007154	cell communication	6558	2121	2.98E-36
GO:0023052	signaling	6532	2111	9.23E-36
GO:0032502	developmental process	6397	2072	1.48E-35
GO:0048522	positive regulation of cellular process	5461	1801	2.26E-34
GO:0045321	leukocyte activation	1267	536	3.83E-34
GO:0044237	cellular metabolic process	11149	3358	4.22E-34
GO:0033036	macromolecule localization	3107	1112	7.13E-34
GO:0048856	anatomical structure development	5974	1942	1.42E-33
GO:0048583	regulation of response to stimulus	4307	1463	3.60E-33
GO:0002250	adaptive immune response	651	319	6.18E-33
GO:0071702	organic substance transport	2784	1005	1.24E-31
GO:0007165	signal transduction	6067	1954	4.60E-31
GO:0008104	protein localization	2757	992	1.66E-30
GO:0044238	primary metabolic process	11117	3320	1.23E-29
GO:0009056	catabolic process	2608	939	1.15E-28
GO:0006950	response to stress	4069	1371	1.18E-28
GO:0051641	cellular localization	2914	1030	1.97E-28
GO:0006614	SRP-dependent cotranslational protein targeting to membrane	103	84	8.92E-28
GO:0070887	cellular response to chemical stimulus	3270	1132	1.28E-27
GO:0016192	vesicle-mediated transport	2089	774	4.24E-27
GO:0008152	metabolic process	12367	3630	7.40E-27

GO:0007275	multicellular organism development	5478	1763	9.50E-27
GO:0002682	regulation of immune system process	1635	631	9.73E-27
GO:0006613	cotranslational protein targeting to membrane	107	85	1.50E-26
GO:0071705	nitrogen compound transport	2367	856	2.27E-26
GO:0019538	protein metabolic process	5938	1889	3.18E-26
GO:0023051	regulation of signaling	3580	1215	5.25E-26
GO:0006928	movement of cell or subcellular component	2206	804	1.12E-25
GO:0051239	regulation of multicellular organismal process	3259	1119	1.24E-25
GO:0048731	system development	4906	1593	2.30E-25
GO:0006807	nitrogen compound metabolic process	10643	3161	5.94E-25
GO:0007166	cell surface receptor signaling pathway	3030	1048	6.18E-25
GO:0046903	secretion	1658	630	1.56E-24
GO:0032940	secretion by cell	1522	587	1.75E-24
GO:0042886	amide transport	2059	754	1.75E-24
GO:0045184	establishment of protein localization	2107	768	2.55E-24
GO:0010033	response to organic substance	3289	1120	4.35E-24
GO:0032879	regulation of localization	2846	989	5.13E-24
GO:0072599	establishment of protein localization to endoplasmic reticulum	120	89	5.95E-24
GO:0045047	protein targeting to ER	116	87	5.98E-24
GO:0009653	anatomical structure morphogenesis	2731	954	7.54E-24
GO:0044248	cellular catabolic process	2317	829	1.13E-23
GO:0015031	protein transport	1992	730	1.17E-23
GO:0015833	peptide transport	2028	741	1.19E-23
GO:0009605	response to external stimulus	2501	884	1.39E-23
GO:0010646	regulation of cell communication	3531	1188	1.41E-23
GO:0035556	intracellular signal transduction	2946	1016	1.75E-23
GO:0070972	protein localization to endoplasmic reticulum	144	100	1.92E-23
GO:0008219	cell death	2254	808	3.07E-23
GO:0006996	organelle organization	3874	1284	5.78E-23
GO:0048869	cellular developmental process	4424	1441	7.67E-23
GO:0032501	multicellular organismal process	7686	2348	1.18E-22
GO:0070727	cellular macromolecule localization	1875	688	3.28E-22
GO:0040011	locomotion	1970	717	3.49E-22
GO:0000184	nuclear-transcribed mRNA catabolic process, nonsense-mediated decay	120	87	3.71E-22
GO:0009966	regulation of signal transduction	3160	1072	4.03E-22
GO:0034613	cellular protein localization	1864	684	4.61E-22
GO:0006915	apoptotic process	1979	719	5.21E-22
GO:0006612	protein targeting to membrane	200	123	6.14E-22
GO:0002443	leukocyte mediated immunity	858	363	6.71E-22
GO:0051128	regulation of cellular component organization	2503	876	9.27E-22
GO:0002252	immune effector process	1249	490	1.13E-21

GO:0012501	programmed cell death	2123	761	1.33E-21
GO:0016477	cell migration	1587	596	1.69E-21
GO:0044085	cellular component biogenesis	3333	1116	6.48E-21
GO:0065009	regulation of molecular function	2977	1011	1.02E-20
GO:0042221	response to chemical	4731	1515	1.10E-20
GO:0072657	protein localization to membrane	619	278	1.24E-20
GO:0022607	cellular component assembly	3095	1044	1.98E-20
GO:1901575	organic substance catabolic process	2169	769	2.61E-20
GO:0048870	cell motility	1744	639	3.88E-20
GO:0051674	localization of cell	1744	639	3.88E-20
GO:0071704	organic substance metabolic process	11910	3458	4.53E-20
GO:0030154	cell differentiation	4233	1369	6.35E-20
GO:0050900	leukocyte migration	495	232	1.05E-19
GO:0042592	homeostatic process	1914	688	1.83E-19
GO:0048584	positive regulation of response to stimulus	2475	856	2.22E-19
GO:0051649	establishment of localization in cell	2234	784	2.37E-19
GO:0030030	cell projection organization	1532	569	4.85E-19
GO:0120036	plasma membrane bounded cell projection organization	1495	557	6.51E-19
GO:0044281	small molecule metabolic process	2107	742	1.56E-18
GO:0046907	intracellular transport	1839	661	1.58E-18
GO:0048513	animal organ development	3575	1172	1.80E-18
GO:0044267	cellular protein metabolic process	5250	1642	7.31E-18
GO:0002274	myeloid leukocyte activation	650	281	7.53E-18
GO:0006793	phosphorus metabolic process	3336	1097	1.99E-17
GO:0050793	regulation of developmental process	2695	909	4.12E-17
GO:0006413	translational initiation	192	112	4.54E-17
GO:0002684	positive regulation of immune system process	1164	446	4.76E-17
GO:0071310	cellular response to organic substance	2699	909	6.73E-17
GO:0048523	negative regulation of cellular process	4885	1533	7.29E-17
GO:0006796	phosphate-containing compound metabolic process	3309	1085	1.01E-16
GO:0010941	regulation of cell death	1728	619	1.06E-16
GO:0043067	regulation of programmed cell death	1607	582	1.13E-16
GO:0048878	chemical homeostasis	1185	451	1.26E-16
GO:0006887	exocytosis	901	360	1.58E-16
GO:0042981	regulation of apoptotic process	1581	573	1.95E-16
GO:0045055	regulated exocytosis	792	324	2.12E-16
GO:0032989	cellular component morphogenesis	1142	436	3.00E-16
GO:0050801	ion homeostasis	809	329	3.18E-16
GO:0090150	establishment of protein localization to membrane	330	164	3.29E-16
GO:0044260	cellular macromolecule metabolic process	8297	2464	3.33E-16
GO:0000902	cell morphogenesis	1033	401	3.93E-16
GO:0006935	chemotaxis	642	273	4.90E-16
GO:0007155	cell adhesion	1417	520	7.93E-16

GO:0042330	taxis	644	273	8.23E-16
GO:0098771	inorganic ion homeostasis	739	304	1.30E-15
GO:0022610	biological adhesion	1424	521	1.47E-15
GO:0050865	regulation of cell activation	599	257	1.57E-15
GO:0055080	cation homeostasis	728	300	1.70E-15
GO:0002366	leukocyte activation involved in immune response	705	292	2.26E-15
GO:0019083	viral transcription	177	103	2.33E-15
GO:0006605	protein targeting	430	198	2.71E-15
GO:0002263	cell activation involved in immune response	709	293	2.85E-15
GO:0043436	oxoacid metabolic process	1168	440	3.49E-15
GO:0023056	positive regulation of signaling	1848	648	3.60E-15
GO:0046649	lymphocyte activation	717	295	4.54E-15
GO:0009058	biosynthetic process	6296	1910	5.27E-15
GO:0019080	viral gene expression	191	108	6.12E-15
GO:0010647	positive regulation of cell communication	1841	644	8.77E-15
GO:1901576	organic substance biosynthetic process	6216	1886	9.50E-15
GO:1901566	organonitrogen compound biosynthetic process	1942	674	9.67E-15
GO:1901700	response to oxygen-containing compound	1633	581	1.03E-14
GO:0002444	myeloid leukocyte mediated immunity	550	238	1.04E-14
GO:0051240	positive regulation of multicellular organismal process	1810	634	1.22E-14
GO:0006082	organic acid metabolic process	1188	444	1.26E-14
GO:0009611	response to wounding	676	280	1.29E-14
GO:0036230	granulocyte activation	503	221	2.22E-14
GO:0043068	positive regulation of programmed cell death	679	280	2.64E-14
GO:0010942	positive regulation of cell death	728	296	2.89E-14
GO:0043065	positive regulation of apoptotic process	671	277	3.41E-14
GO:0050790	regulation of catalytic activity	2322	782	6.49E-14
GO:0002275	myeloid cell activation involved in immune response	543	233	8.35E-14
GO:0006886	intracellular protein transport	1113	417	9.63E-14
GO:0042119	neutrophil activation	497	217	1.10E-13
GO:0051270	regulation of cellular component movement	1096	411	1.42E-13
GO:0030097	hemopoiesis	887	345	1.42E-13
GO:0007264	small GTPase mediated signal transduction	569	241	1.52E-13
GO:0042110	T cell activation	464	205	1.91E-13
GO:0007010	cytoskeleton organization	1329	482	2.57E-13
GO:0044249	cellular biosynthetic process	6125	1849	2.84E-13
GO:0043299	leukocyte degranulation	533	228	2.97E-13
GO:0048534	hematopoietic or lymphoid organ development	929	357	3.30E-13
GO:0006811	ion transport	1691	590	5.78E-13
GO:0009967	positive regulation of signal transduction	1682	587	6.64E-13
GO:0042060	wound healing	563	237	6.87E-13
GO:0002446	neutrophil mediated immunity	498	215	7.29E-13
GO:0006464	cellular protein modification process	4147	1297	8.66E-13

GO:0036211	protein modification process	4147	1297	8.66E-13
GO:0002520	immune system development	982	372	9.28E-13
GO:0002694	regulation of leukocyte activation	556	234	1.13E-12
GO:0019752	carboxylic acid metabolic process	1079	402	1.14E-12
GO:0051049	regulation of transport	1875	643	1.33E-12
GO:0000956	nuclear-transcribed mRNA catabolic process	207	110	1.42E-12
GO:0006873	cellular ion homeostasis	663	269	1.52E-12
GO:0030334	regulation of cell migration	950	361	1.59E-12
GO:0006629	lipid metabolic process	1404	501	1.83E-12
GO:0051704	multi-organism process	2614	858	2.26E-12
GO:0019725	cellular homeostasis	968	366	2.35E-12
GO:0060326	cell chemotaxis	302	145	2.51E-12
GO:0030003	cellular cation homeostasis	650	264	2.54E-12
GO:0097190	apoptotic signaling pathway	602	248	2.93E-12
GO:0044403	symbiont process	881	338	3.24E-12
GO:0002283	neutrophil activation involved in immune response	487	209	4.39E-12
GO:2000145	regulation of cell motility	1011	378	5.43E-12
GO:0043312	neutrophil degranulation	484	207	9.49E-12
GO:0016032	viral process	830	320	9.70E-12
GO:2000026	regulation of multicellular organismal development	2156	721	9.77E-12
GO:0048519	negative regulation of biological process	5841	1758	1.02E-11
GO:0048468	cell development	2181	728	1.11E-11
GO:0040012	regulation of locomotion	1083	399	1.16E-11
GO:0055065	metal ion homeostasis	651	262	1.26E-11
GO:0016310	phosphorylation	2409	794	1.28E-11
GO:0043412	macromolecule modification	4358	1347	1.37E-11
GO:0050776	regulation of immune response	1091	401	1.50E-11
GO:0008283	cell proliferation	2021	680	1.73E-11
GO:0009893	positive regulation of metabolic process	3631	1141	2.63E-11
GO:0055082	cellular chemical homeostasis	810	312	2.64E-11
GO:0051336	regulation of hydrolase activity	1292	461	3.42E-11
GO:0044419	interspecies interaction between organisms	929	349	3.62E-11
GO:0042127	regulation of cell proliferation	1697	582	4.57E-11
GO:0006952	defense response	1767	602	6.31E-11
GO:0072594	establishment of protein localization to organelle	546	225	7.30E-11
GO:0030595	leukocyte chemotaxis	222	112	9.72E-11
GO:0033365	protein localization to organelle	918	343	1.53E-10
GO:0034097	response to cytokine	1205	431	1.94E-10
GO:0007399	nervous system development	2371	775	2.50E-10
GO:0051050	positive regulation of transport	980	361	3.10E-10
GO:0030029	actin filament-based process	758	291	3.97E-10
GO:0001816	cytokine production	790	301	4.29E-10
GO:0055085	transmembrane transport	1594	546	4.68E-10

GO:0051094	positive regulation of developmental process	1417	493	5.07E-10
GO:0045595	regulation of cell differentiation	1878	629	6.53E-10
GO:0098657	import into cell	931	344	8.20E-10
GO:0043062	extracellular structure organization	422	180	1.05E-09
GO:0032101	regulation of response to external stimulus	910	337	1.06E-09
GO:0051249	regulation of lymphocyte activation	470	196	1.13E-09
GO:0010035	response to inorganic substance	561	226	1.13E-09
GO:0098609	cell-cell adhesion	839	314	1.71E-09
GO:0072507	divalent inorganic cation homeostasis	512	209	2.17E-09
GO:0044093	positive regulation of molecular function	1767	593	2.55E-09
GO:0009057	macromolecule catabolic process	1391	481	3.33E-09
GO:0051186	cofactor metabolic process	597	236	3.47E-09
GO:1901701	cellular response to oxygen-containing compound	1133	403	3.80E-09
GO:1901361	organic cyclic compound catabolic process	745	283	3.97E-09
GO:0042493	response to drug	1019	368	4.24E-09
GO:0006897	endocytosis	806	302	4.37E-09
GO:0006875	cellular metal ion homeostasis	577	229	4.91E-09
GO:0030335	positive regulation of cell migration	544	218	5.94E-09
GO:0040017	positive regulation of locomotion	597	235	6.61E-09
GO:0030001	metal ion transport	880	324	7.77E-09
GO:0031323	regulation of cellular metabolic process	6268	1848	7.92E-09
GO:0006954	inflammatory response	842	312	8.66E-09
GO:0065003	protein-containing complex assembly	1951	643	1.09E-08
GO:1902531	regulation of intracellular signal transduction	1958	645	1.10E-08
GO:0051272	positive regulation of cellular component movement	584	230	1.11E-08
GO:0046700	heterocycle catabolic process	695	265	1.44E-08
GO:0048585	negative regulation of response to stimulus	1690	566	1.45E-08
GO:0007599	hemostasis	341	149	1.63E-08
GO:0048646	anatomical structure formation involved in morphogenesis	1170	411	1.73E-08
GO:0019439	aromatic compound catabolic process	712	270	1.79E-08
GO:0000904	cell morphogenesis involved in differentiation	744	280	1.86E-08
GO:0030036	actin cytoskeleton organization	665	255	1.94E-08
GO:0031175	neuron projection development	977	352	2.05E-08
GO:0010604	positive regulation of macromolecule metabolic process	3357	1042	2.05E-08
GO:0034220	ion transmembrane transport	1178	413	2.09E-08
GO:0007265	Ras protein signal transduction	446	184	2.11E-08
GO:0032880	regulation of protein localization	1010	362	2.14E-08
GO:0006790	sulfur compound metabolic process	369	158	2.50E-08
GO:0031325	positive regulation of cellular metabolic process	3321	1031	2.66E-08
GO:0002685	regulation of leukocyte migration	196	97	2.83E-08
GO:0051130	positive regulation of cellular component organization	1257	436	2.92E-08
GO:2000147	positive regulation of cell motility	567	223	2.94E-08
GO:0043603	cellular amide metabolic process	1144	402	2.95E-08

GO:0022008	neurogenesis	1622	544	3.23E-08
GO:0051247	positive regulation of protein metabolic process	1708	569	3.53E-08
GO:0030155	regulation of cell adhesion	691	262	3.99E-08
GO:0072503	cellular divalent inorganic cation homeostasis	492	198	4.62E-08
GO:0007596	blood coagulation	336	146	4.63E-08
GO:0044255	cellular lipid metabolic process	1068	378	4.74E-08
GO:0009636	response to toxic substance	523	208	4.88E-08
GO:0006812	cation transport	1155	404	5.40E-08
GO:0044270	cellular nitrogen compound catabolic process	696	263	5.55E-08
GO:0008360	regulation of cell shape	150	79	5.87E-08
GO:0001817	regulation of cytokine production	719	270	6.32E-08
GO:0071345	cellular response to cytokine stimulus	1112	390	8.71E-08
GO:0070661	leukocyte proliferation	298	132	1.01E-07
GO:0050817	coagulation	342	147	1.06E-07
GO:0006979	response to oxidative stress	450	183	1.08E-07
GO:0006468	protein phosphorylation	1980	645	1.11E-07
GO:0002521	leukocyte differentiation	515	204	1.23E-07
GO:0051174	regulation of phosphorus metabolic process	1812	596	1.24E-07
GO:0050878	regulation of body fluid levels	500	199	1.34E-07
GO:1901135	carbohydrate derivative metabolic process	1259	433	1.41E-07
GO:0019220	regulation of phosphate metabolic process	1810	595	1.44E-07
GO:0050863	regulation of T cell activation	314	137	1.53E-07
GO:1901565	organonitrogen compound catabolic process	1290	442	1.56E-07
GO:0030031	cell projection assembly	566	220	1.59E-07
GO:0045597	positive regulation of cell differentiation	991	352	1.66E-07
GO:0051173	positive regulation of nitrogen compound metabolic process	3191	988	1.74E-07
GO:0055114	oxidation-reduction process	992	352	1.92E-07
GO:0030198	extracellular matrix organization	368	155	1.93E-07
GO:0050778	positive regulation of immune response	881	318	2.05E-07
GO:0051046	regulation of secretion	810	296	2.22E-07
GO:0061024	membrane organization	875	316	2.22E-07
GO:0048666	neuron development	1109	387	2.26E-07
GO:0010648	negative regulation of cell communication	1416	478	2.72E-07
GO:0034655	nucleobase-containing compound catabolic process	648	245	2.92E-07
GO:0023057	negative regulation of signaling	1420	479	2.92E-07
GO:0055074	calcium ion homeostasis	470	188	3.08E-07
GO:0050867	positive regulation of cell activation	379	158	3.19E-07
GO:0022603	regulation of anatomical structure morphogenesis	1155	400	3.25E-07
GO:0051246	regulation of protein metabolic process	2923	910	3.75E-07
GO:0080090	regulation of primary metabolic process	6195	1810	4.86E-07
GO:0048699	generation of neurons	1523	508	4.86E-07
GO:0080134	regulation of response to stress	1636	541	4.95E-07
GO:0044282	small molecule catabolic process	445	179	5.45E-07

GO:0042325	regulation of phosphorylation	1610	533	5.78E-07
GO:0120031	plasma membrane bounded cell projection assembly	554	214	5.99E-07
GO:0009968	negative regulation of signal transduction	1311	445	6.03E-07
GO:0045785	positive regulation of cell adhesion	403	165	6.34E-07
GO:0002449	lymphocyte mediated immunity	337	143	6.38E-07
GO:0032270	positive regulation of cellular protein metabolic process	1606	531	7.89E-07
GO:0042327	positive regulation of phosphorylation	1066	371	9.02E-07
GO:0071496	cellular response to external stimulus	339	143	1.06E-06
GO:0009888	tissue development	2062	662	1.07E-06
GO:0032787	monocarboxylic acid metabolic process	649	243	1.08E-06
GO:0005975	carbohydrate metabolic process	624	235	1.18E-06
GO:0002237	response to molecule of bacterial origin	343	144	1.38E-06
GO:0006402	mRNA catabolic process	364	151	1.38E-06
GO:0043933	protein-containing complex subunit organization	2270	720	1.73E-06
GO:1903530	regulation of secretion by cell	752	274	1.86E-06
GO:0071216	cellular response to biotic stimulus	236	107	1.93E-06
GO:0070663	regulation of leukocyte proliferation	222	102	2.06E-06
GO:0043087	regulation of GTPase activity	476	187	2.09E-06
GO:0043085	positive regulation of catalytic activity	1420	474	2.10E-06
GO:0048858	cell projection morphogenesis	666	247	2.23E-06
GO:2001233	regulation of apoptotic signaling pathway	406	164	2.51E-06
GO:0070925	organelle assembly	842	301	2.52E-06
GO:0022407	regulation of cell-cell adhesion	403	163	2.54E-06
GO:1903037	regulation of leukocyte cell-cell adhesion	304	130	2.68E-06
GO:0008610	lipid biosynthetic process	706	259	2.80E-06
GO:0007159	leukocyte cell-cell adhesion	337	141	2.88E-06
GO:0035295	tube development	1110	381	3.60E-06
GO:0032990	cell part morphogenesis	685	252	3.63E-06
GO:0006874	cellular calcium ion homeostasis	457	180	3.65E-06
GO:0043170	macromolecule metabolic process	10333	2904	3.69E-06
GO:0030182	neuron differentiation	1364	456	3.83E-06
GO:0001934	positive regulation of protein phosphorylation	1015	352	4.65E-06
GO:0032496	response to lipopolysaccharide	330	138	4.70E-06
GO:0044092	negative regulation of molecular function	1156	394	4.81E-06
GO:0120039	plasma membrane bounded cell projection morphogenesis	662	244	5.75E-06
GO:0010562	positive regulation of phosphorus metabolic process	1138	388	6.31E-06
GO:0045937	positive regulation of phosphate metabolic process	1138	388	6.31E-06
GO:0060548	negative regulation of cell death	1018	352	6.89E-06
GO:0002696	positive regulation of leukocyte activation	365	149	7.27E-06
GO:0006914	autophagy	495	191	7.64E-06
GO:0061919	process utilizing autophagic mechanism	495	191	7.64E-06
GO:0071219	cellular response to molecule of bacterial origin	212	97	7.76E-06
GO:0051345	positive regulation of hydrolase activity	768	276	8.13E-06

GO:0051223	regulation of protein transport	697	254	8.90E-06
GO:0032268	regulation of cellular protein metabolic process	2668	827	9.39E-06
GO:0040007	growth	984	341	9.83E-06
GO:0009887	animal organ morphogenesis	1068	366	1.03E-05
GO:0001909	leukocyte mediated cytotoxicity	107	58	1.04E-05
GO:0002764	immune response-regulating signaling pathway	656	241	1.05E-05
GO:0002790	peptide secretion	624	231	1.09E-05
GO:0030099	myeloid cell differentiation	416	165	1.13E-05
GO:0009719	response to endogenous stimulus	1656	538	1.18E-05
GO:1902533	positive regulation of intracellular signal transduction	1080	369	1.31E-05
GO:0097529	myeloid leukocyte migration	208	95	1.32E-05
GO:0072511	divalent inorganic cation transport	489	188	1.47E-05
GO:0030217	T cell differentiation	240	106	1.50E-05
GO:0031344	regulation of cell projection organization	681	248	1.54E-05
GO:0070838	divalent metal ion transport	483	186	1.56E-05
GO:0009306	protein secretion	591	220	1.60E-05
GO:0002181	cytoplasmic translation	98	54	1.83E-05
GO:0044283	small molecule biosynthetic process	803	285	1.83E-05
GO:0070201	regulation of establishment of protein localization	744	267	1.84E-05
GO:0022604	regulation of cell morphogenesis	484	186	1.88E-05
GO:0033993	response to lipid	923	321	2.07E-05
GO:0002768	immune response-regulating cell surface receptor signaling pathway	488	187	2.22E-05
GO:0048812	neuron projection morphogenesis	648	237	2.34E-05
GO:0032943	mononuclear cell proliferation	274	117	2.45E-05
GO:0120035	regulation of plasma membrane bounded cell projection organization	671	244	2.47E-05
GO:0033043	regulation of organelle organization	1258	420	2.50E-05
GO:0051171	regulation of nitrogen compound metabolic process	6025	1745	2.58E-05
GO:0043069	negative regulation of programmed cell death	928	322	2.59E-05
GO:0044271	cellular nitrogen compound biosynthetic process	4989	1464	2.61E-05
GO:0090087	regulation of peptide transport	727	261	2.72E-05
GO:0009059	macromolecule biosynthetic process	5077	1487	3.20E-05
GO:0018193	peptidyl-amino acid modification	1257	419	3.28E-05
GO:0009617	response to bacterium	683	247	3.51E-05
GO:0051047	positive regulation of secretion	428	167	3.72E-05
GO:0006401	RNA catabolic process	397	157	3.75E-05
GO:0002460	adaptive immune response based on somatic recombination of immune receptors built from immunoglobulin superfamily domains	346	140	4.90E-05
GO:0031668	cellular response to extracellular stimulus	268	114	5.21E-05
GO:0043066	negative regulation of apoptotic process	907	314	5.46E-05
GO:0050707	regulation of cytokine secretion	210	94	5.87E-05

GO:0098655	cation transmembrane transport	851	297	5.89E-05
GO:0044265	cellular macromolecule catabolic process	1157	388	6.08E-05
GO:0046651	lymphocyte proliferation	272	115	6.79E-05
GO:0009607	response to biotic stimulus	1030	350	7.30E-05
GO:0071222	cellular response to lipopolysaccharide	205	92	7.34E-05
GO:0050663	cytokine secretion	240	104	7.72E-05
GO:0019222	regulation of metabolic process	7252	2069	7.76E-05
GO:0046677	response to antibiotic	327	133	8.29E-05
GO:0022409	positive regulation of cell-cell adhesion	255	109	8.33E-05
GO:0050920	regulation of chemotaxis	217	96	8.42E-05
GO:0034612	response to tumor necrosis factor	309	127	8.68E-05
GO:0010038	response to metal ion	364	145	8.79E-05
GO:0050851	antigen receptor-mediated signaling pathway	300	124	8.80E-05
GO:0031399	regulation of protein modification process	1862	591	9.00E-05
GO:0032944	regulation of mononuclear cell proliferation	209	93	0.000102
GO:0048667	cell morphogenesis involved in neuron differentiation	583	214	0.000106
GO:0002757	immune response-activating signal transduction	622	226	0.000109
GO:0031401	positive regulation of protein modification process	1234	409	0.000112
GO:0002253	activation of immune response	704	251	0.000116
GO:0035239	tube morphogenesis	920	316	0.000123
GO:0006732	coenzyme metabolic process	401	156	0.00016
GO:0050670	regulation of lymphocyte proliferation	208	92	0.000178
GO:0001932	regulation of protein phosphorylation	1462	474	0.000179
GO:1903532	positive regulation of secretion by cell	399	155	0.000199
GO:0002683	negative regulation of immune system process	462	175	0.000202
GO:0051251	positive regulation of lymphocyte activation	319	129	0.000214
GO:0050708	regulation of protein secretion	472	178	0.00022
GO:0007266	Rho protein signal transduction	203	90	0.000224
GO:0002456	T cell mediated immunity	106	55	0.000232
GO:0002688	regulation of leukocyte chemotaxis	114	58	0.000239
GO:1903039	positive regulation of leukocyte cell-cell adhesion	218	95	0.000255
GO:1901137	carbohydrate derivative biosynthetic process	769	269	0.000258
GO:0051222	positive regulation of protein transport	416	160	0.000259
GO:0001819	positive regulation of cytokine production	464	175	0.00029
GO:0043207	response to external biotic stimulus	998	337	0.000303
GO:0072359	circulatory system development	1168	387	0.000305
GO:0044087	regulation of cellular component biogenesis	951	323	0.000312
GO:0042098	T cell proliferation	184	83	0.000314
GO:0006508	proteolysis	1856	585	0.000335
GO:0034645	cellular macromolecule biosynthetic process	4924	1434	0.000336
GO:0006909	phagocytosis	355	140	0.000341
GO:0031589	cell-substrate adhesion	352	139	0.000348
GO:0051707	response to other organism	996	336	0.000357

GO:0002228	natural killer cell mediated immunity	64	38	0.000366
GO:0046394	carboxylic acid biosynthetic process	459	173	0.000369
GO:0050866	negative regulation of cell activation	199	88	0.000376
GO:0010256	endomembrane system organization	437	166	0.000382
GO:0050870	positive regulation of T cell activation	202	89	0.000387
GO:0030098	lymphocyte differentiation	353	139	0.000428
GO:0016053	organic acid biosynthetic process	460	173	0.000441
GO:0001906	cell killing	168	77	0.000442
GO:0045087	innate immune response	981	331	0.000447
GO:0010506	regulation of autophagy	326	130	0.000506
GO:0006820	anion transport	629	225	0.000522
GO:0008284	positive regulation of cell proliferation	949	321	0.000552
GO:0002429	immune response-activating cell surface receptor signaling pathway	455	171	0.000562
GO:1901698	response to nitrogen compound	1085	361	0.000571
GO:0061564	axon development	513	189	0.0006
GO:0016054	organic acid catabolic process	275	113	0.000615
GO:0046395	carboxylic acid catabolic process	275	113	0.000615
GO:0097435	supramolecular fiber organization	670	237	0.00066
GO:0019221	cytokine-mediated signaling pathway	793	274	0.00069
GO:0006816	calcium ion transport	434	164	0.000702
GO:0031669	cellular response to nutrient levels	237	100	0.00079
GO:0050715	positive regulation of cytokine secretion	139	66	0.000824
GO:0098662	inorganic cation transmembrane transport	748	260	0.000855
GO:0001944	vasculature development	792	273	0.000934
GO:0007267	cell-cell signaling	1647	522	0.000948
GO:0002791	regulation of peptide secretion	500	184	0.001
GO:0007409	axonogenesis	468	174	0.001019
GO:0010243	response to organonitrogen compound	995	333	0.001031
GO:0009894	regulation of catabolic process	975	327	0.001075
GO:1903706	regulation of hemopoiesis	475	176	0.001127
GO:0072358	cardiovascular system development	801	275	0.001255
GO:0009628	response to abiotic stimulus	1233	402	0.00126
GO:0090066	regulation of anatomical structure size	505	185	0.001337
GO:0044272	sulfur compound biosynthetic process	189	83	0.001372
GO:1904951	positive regulation of establishment of protein localization	454	169	0.001436
GO:0043086	negative regulation of catalytic activity	813	278	0.001601
GO:0009100	glycoprotein metabolic process	417	157	0.001795
GO:0006575	cellular modified amino acid metabolic process	199	86	0.001933
GO:0019637	organophosphate metabolic process	1172	383	0.001974
GO:0030168	platelet activation	153	70	0.002073
GO:0043547	positive regulation of GTPase activity	402	152	0.0021
GO:0015711	organic anion transport	482	177	0.002113

GO:0070665	positive regulation of leukocyte proliferation	139	65	0.002143
GO:0002687	positive regulation of leukocyte migration	128	61	0.002251
GO:1903708	positive regulation of hemopoiesis	185	81	0.002298
GO:0051259	protein complex oligomerization	567	203	0.002306
GO:0008285	negative regulation of cell proliferation	759	261	0.002322
GO:0021700	developmental maturation	284	114	0.00238
GO:0001525	angiogenesis	587	209	0.002428
GO:0032946	positive regulation of mononuclear cell proliferation	131	62	0.002435
GO:0060341	regulation of cellular localization	901	303	0.002505
GO:0051480	regulation of cytosolic calcium ion concentration	356	137	0.002694
GO:0055067	monovalent inorganic cation homeostasis	154	70	0.002839
GO:0001568	blood vessel development	761	261	0.002991
GO:0030203	glycosaminoglycan metabolic process	160	72	0.003097
GO:0098754	detoxification	129	61	0.003192
GO:0017144	drug metabolic process	846	286	0.003351
GO:0045926	negative regulation of growth	249	102	0.003394
GO:0000302	response to reactive oxygen species	231	96	0.003478
GO:0090382	phagosome maturation	44	28	0.003566
GO:0050921	positive regulation of chemotaxis	135	63	0.003671
GO:0009790	embryo development	1017	336	0.003783
GO:2001236	regulation of extrinsic apoptotic signaling pathway	155	70	0.003867
GO:0050671	positive regulation of lymphocyte proliferation	130	61	0.004496
GO:0098660	inorganic ion transmembrane transport	832	281	0.004715
GO:1902105	regulation of leukocyte differentiation	272	109	0.004932
GO:0002697	regulation of immune effector process	455	167	0.004978
GO:0033554	cellular response to stress	2060	633	0.005123
GO:0042129	regulation of T cell proliferation	156	70	0.005243
GO:0009101	glycoprotein biosynthetic process	347	133	0.005324
GO:0007169	transmembrane receptor protein tyrosine kinase signaling pathway	719	247	0.005533
GO:0042102	positive regulation of T cell proliferation	95	48	0.005595
GO:0071356	cellular response to tumor necrosis factor	288	114	0.005613
GO:2001234	negative regulation of apoptotic signaling pathway	230	95	0.005645
GO:0097191	extrinsic apoptotic signaling pathway	224	93	0.005674
GO:1901699	cellular response to nitrogen compound	646	225	0.005774
GO:0009991	response to extracellular stimulus	531	190	0.006086
GO:0010628	positive regulation of gene expression	2009	618	0.006115
GO:0006885	regulation of pH	98	49	0.006362
GO:0048608	reproductive structure development	431	159	0.006652
GO:1990778	protein localization to cell periphery	311	121	0.007268
GO:0051338	regulation of transferase activity	972	321	0.007418
GO:1901360	organic cyclic compound metabolic process	6715	1899	0.007434
GO:0043549	regulation of kinase activity	870	291	0.007605

GO:0002695	negative regulation of leukocyte activation	175	76	0.008302
GO:0060284	regulation of cell development	953	315	0.008722
GO:0044242	cellular lipid catabolic process	217	90	0.00926
GO:0043604	amide biosynthetic process	865	289	0.009313
GO:0042267	natural killer cell mediated cytotoxicity	60	34	0.009762
GO:0048729	tissue morphogenesis	670	231	0.009801
GO:0032103	positive regulation of response to external stimulus	322	124	0.009964

Table S3. Comprehensive list of all statistically significant GSEA genesets enriched in RNA-sequencing data. A total of 31 subsets from the Hallmarks geneset were enriched in CTCs compared to HDs. A false-discovery rate (FDR) < 0.25 and nominal p-value < 0.05 were used.

NAME	SIZE	NES	NOM p-val	FDR q-val
HALLMARK_PROTEIN_SECRETION	96	2.946136	0	0
HALLMARK_MTORC1_SIGNALING	200	2.833126	0	0
HALLMARK_CHOLESTEROL_HOMEOSTASIS	73	2.609091	0	0
HALLMARK_OXIDATIVE_PHOSPHORYLATION	200	2.604375	0	0
HALLMARK_ADIPOGENESIS	199	2.55849	0	0
HALLMARK_XENOBIOTIC_METABOLISM	191	2.533498	0	0
HALLMARK_FATTY_ACID_METABOLISM	151	2.43049	0	0
HALLMARK_GLYCOLYSIS	198	2.42551	0	0
HALLMARK_PEROXISOME	100	2.218617	0	0
HALLMARK_ANDROGEN_RESPONSE	98	2.213071	0	0
HALLMARK_TNFA_SIGNALING_VIA_NFKB	200	2.182778	0	0
HALLMARK_COAGULATION	128	2.154189	0	0
HALLMARK_HYPOXIA	194	2.144452	0	0
HALLMARK_UV_RESPONSE_UP	155	2.09599	0	0
HALLMARK_EPITHELIAL_MESENCHYMAL_TRANSITION	196	2.03004	0	0
HALLMARK_COMPLEMENT	200	1.825486	0	9.41E-04
HALLMARK_P53_PATHWAY	198	1.791781	0	0.001295
HALLMARK_MYOGENESIS	189	1.784504	0	0.001223
HALLMARK_ANGIOGENESIS	33	1.736989	0.005587	0.001775
HALLMARK_TGF_BETA_SIGNALING	54	1.691478	0.005634	0.002713
HALLMARK_UNFOLDED_PROTEIN_RESPONSE	112	1.666025	0.003175	0.003437
HALLMARK_ESTROGEN_RESPONSE_EARLY	193	1.596673	0	0.007607
HALLMARK_APOPTOSIS	161	1.563398	0	0.010004
HALLMARK_BILE_ACID_METABOLISM	106	1.544936	0.006623	0.011672
HALLMARK_INFLAMMATORY_RESPONSE	196	1.543884	0.003984	0.011205
HALLMARK_ESTROGEN_RESPONSE_LATE	190	1.51786	0	0.013228
HALLMARK_IL2_STAT5_SIGNALING	200	1.507252	0	0.01409
HALLMARK_APICAL_JUNCTION	195	1.48812	0	0.015856
HALLMARK_REACTIVE_OXYGEN_SPECIES_PATHWAY	49	1.433821	0.044693	0.02322
HALLMARK_PI3K_AKT_MTOR_SIGNALING	102	1.332461	0.032641	0.050312
HALLMARK_IL6_JAK_STAT3_SIGNALING	85	1.329312	0.037855	0.049944

Table S4. Primers used in this study.

Gene	Accession Number	Forward Primer (5′-3′)	Reverse Primer (5′-3′)
CK5	NM_000424.4	TCAGACCAAGTATGAGGAGC	GACAACAGAGATGTTGACTG
CK8	NM_002273.4	CATAGACAAGGTACGGTTCC	CTTCATCCACATCCTTCTTG
VIM	NM_003380.5	CCTGGATTTCCTCTTCGTGG	TCCGGGAGAAATTGCAGGAG
SERPINE1	NM_000602.5	GTGGACTTTTCAGAGGTGGAG	TGATTTGTGGAAGAGGCGG
SCG2A2 (MAM)	NM_002411.4	CTCCCAGCACTGCTACGCAGGCTC	CACCTCAACATTGCTCAGAGTTTCATCCG
CXCR2	NM_001557.4	CCCTCATCTACGCCTTCATTG	TGAGGCTTGGAATGTGACTG
SNAI1	NM_005985.4	ACCCCAATCGGAAGCCTAAC	AGCCTTTCCCACTGTCCTCA
ESR1	NM_000125.4	AGGGAAAATGTGTAGAGGGC	AGGTGGATCAAAGTGTCTGTG
PGR	NM_000926.4	GGTGTTTGGTCTAGGATGGAG	ACTGGGTTTGACTTCGTAGC
ERBB2	NM_001005862.2	GAGACCCGCTGAACAATACC	CTTACACATCGGAGAACAGGG
GAPDH	NM_002046.7	AATCCCATCACCATCTTCCAG	GTTGTCATACTTCTCATGGTTCAC
CD45	NM_002838.5	AGGGAACAAGCATCACAAGAG	CATTCTGAGCAGGGTAGGTG
RHOD	NM_014578.4	TGCTGATGGTCTTCGCCGAT	CATAGTCATCTTGCCCTGCTGT
CCND1	NM_053056.3	GATCAAGTGTGACCCGGACT	CTTGGGGTCCATGTTCTGCT
CDKN1A	NM_000389.5	GACTGTGATGCGCTAATGGC	CGTTTGGAGTGGTAGAAATCTGTC
BCAT1	NM_005504.7	CGCCACGTGTACTCGCC	TCCGTTACTGCAATCCTTCATT
TGM2	NM_004613.4	ATGGCCGAGGAGCTGGT	CAGCACAAAGCTGGATCCCT
ENG	NM_001114753.3	TCACTGCCATCCATTGGAGC	TGTTTCTGCAAGACTTGTGGG
PHLDA3	NM_012396.5	CTGAGGTCAAGGCAGCTTCG	GGGCATGTCCTGAGAGTCTG

cancers

MDPI

Supplemental References

1. Hensler, M.; Vancurova, I.; Becht, E.; Palata, O.; Strnad, P.; Tesarova, P.; Cabinakova, M.; Svec, D.; Kubista, M.; Bartunkova, J.; et al. Gene expression profiling of circulating tumor cells and peripheral blood mononuclear cells from breast cancer patients. *OncoImmunology* **2015**, *5*, e1102827, doi:10.1080/2162402x.2015.1102827.

© 2020 by the authors. Submitted for possible open access publication under the terms and conditions of the Creative Commons Attribution (CC BY) license (http://creativecommons.org/licenses/by/4.0/).
